# Cypripedin diminishes an epithelial-to-mesenchymal transition in non-small cell lung cancer cells through suppression of Akt/GSK-3β signalling

**DOI:** 10.1038/s41598-018-25657-5

**Published:** 2018-05-22

**Authors:** Surassawadee Treesuwan, Boonchoo Sritularak, Pithi Chanvorachote, Varisa Pongrakhananon

**Affiliations:** 1 Cell-Based Drug and Health Product Development Research Unit, Chulalongkorn University, Bangkok, 10330 Thailand; 2 Department of Pharmacology and Physiology, Faculty of Pharmaceutical Sciences, Chulalongkorn University, Bangkok, 10330 Thailand; 3 Department of Pharmacognosy and Pharmaceutical Botany, Faculty of Pharmaceutical Sciences, Chulalongkorn University, Bangkok, 10330 Thailand

## Abstract

Lung cancer appears to have the highest rate of mortality among cancers due to its metastasis capability. To achieve metastasis, cancer cells acquire the ability to undergo a switch from epithelial to mesenchymal behaviour, termed the epithelial-to-mesenchymal transition (EMT), which is associated with poor clinical outcomes. Drug discovery attempts have been made to find potent compounds that will suppress EMT. Cypripedin, a phenanthrenequinone isolated from Thai orchid, *Dendrobium densiflorum*, exhibits diverse pharmacological activities. In this study, we found that cypripedin attenuated typical mesenchymal phenotypes, including migratory behaviour, of non-small cell lung cancer H460 cells, with a significant reduction of actin stress fibres and focal adhesion and with weakened anchorage-independent growth. Western blot analysis revealed that the negative activity of this compound on EMT was a result of the down-regulation of the EMT markers Slug, N-Cadherin and Vimentin, which was due to ATP-dependent tyrosine kinase (Akt) inactivation. As a consequence, the increase in the Slug degradation rate via a ubiquitin-proteasomal mechanism was encouraged. The observation in another lung cancer H23 cell line also supported this finding, indicating that cypripedin exhibits a promising pharmacological action on lung cancer metastasis that could provide scientific evidence for the further development of this compound.

## Introduction

Lung cancer has become the leading cause of cancer death, and the number of deaths has been gradually increasing every year. Most lung cancer pateints are diagnosed at an advanced stage, for which the 5 year-survival rate after detection is considerably lower^1^. The suppression of cancer metastasis could improve clinical outcomes. Cancer metastasis is defined as a spread of cancer cells from the primary site of origin to distant organs, and it is an important hallmark characteristic of cancer^2^. Cancer cells spread throughout the body in a series of integrated-steps, of which the transition from epithelial to mesenchymal activity plays a crucial prerequisite phase that enhances the motile ability and survival of metastatic cells^3^.

These trans-differentiation features are involved in the loss of cell-cell adhesion and with the morphological change to spindle-like mesenchymal cells that are favourable for migration^4^. The up-regulation of N-Cadherin (N-Cad) as opposed to E-Cadherin (E-Cad), termed Cadherin switching, is regulated by the transcription factor Slug and leads to cell dissociation^5,6^. It has been reported that the inhibition of Slug expression extensively attenuated ovarian cancer metastasis both for *in vitro* and *in vivo* experiments^7^. In addition, Vimentin, an intermediate filament that is essential for mesenchymal cell movement, was increased^8^. The aberrant expressions of these mesenchymal regulatory proteins contributed to the non-responsiveness of clinical outcomes to treatments^9–11^ and are attractive targets for curative strategy.

**Figure 1:**
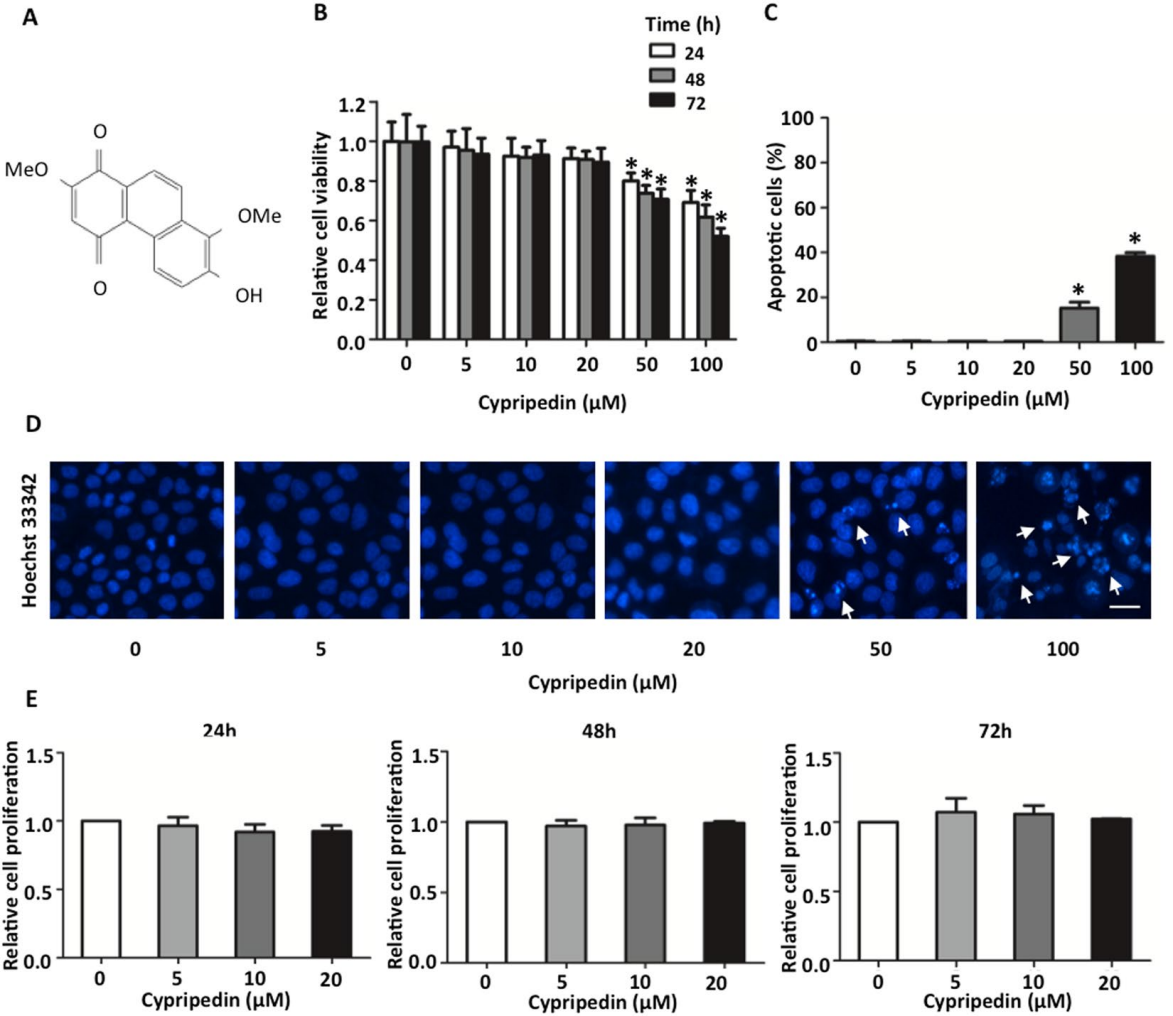
Cytotoxicity of cypripedin on lung cancer H460 cells. (**A**) Chemical structure of cypripedin. (**B**) H460 cells were treated with various concentrations (0–100 µM) of cypripedin for 24, 48 and 72 h; cell viability was measured by MTT assay and is represented as a mean of the relative value. The data are presented as mean ± SEM (n = 4). *p < 0.05 compared with untreated control cells. (**C**) H460 cells were treated with various concentrations (0–100 µM) of cypripedin for 72 h; apoptotic cells were investigated by Hoechst 33342 nuclear staining dye. The percentage of apoptotic cells was calculated comparing to the control cells. The data are presented as mean ± SEM (n = 4). *p < 0.05 compared with control cells. (**D**) The cells with apoptotic nuclei are illustrated (scale bar is 10 µm). (**E**) H460 cells were treated with non-toxic concentrations (0–20 µM) of cypripedin for 24, 48 and 72 h; cell growth was examined by cell proliferation assay and is presented as a relative value. The data are presented as mean ± SEM (n = 4). *p < 0.05 compared with untreated control cells.

At present, the current therapy available was not able to suppress cancer metastasis, even though the diagnostic technology and therapeutic interventions were greatly improved^12^. The discovery of new substances provides an alternative approach to eliminate metastasized cancer cells. The compounds derived from plants have been long used as an alternative therapy, including the substances from orchids. *Dendrobium densiflorum*, a member of the *Dendrobium* species, is the source of several biological compounds, including cypripedin, gigantol, moscatilin, tristin, naringenin and homoeriodictyol^13^. Previous studies indicated that the phenolic compounds from this orchid pose anti-cancer properties in various tumour types, including growth inhibition^14,15^, exertion of apoptosis^16,17^ and inhibition of cell migration and invasion^18–20^. Cypripedin (Fig. 1A), a phenanthrenequinone isolated from this plant, also exhibited numerous pharmacological activities, such as anti-spasmodic, sedative, diaphoretic, hypnotic, and anxiolytic properties^21^. However, its anti-metastasis effects were not reported. Since EMT is a primary process required for cancer metastasis, this study aimed to examine whether cypripedin was able to attenuate this aggressive behaviour in *in vitro* lung cancer cells and to examine the underlying mechanism.

## Results

### Cytotoxicity of cypripedin on lung cancer H460 cells

To diminish the interference of cytotoxic effect of cypripedin on cell motility, we first investigated the effect of this compound on H460 cell death using the MTT assay. H460 cells were treated with various concentrations of cypripedin (0–100 µM) for 24, 48 and 72 h. The results demonstrated that after cypripedin treatment for 24 h, the cell viability was reduced to 0.79- and 0.69-fold for 50 and 100 µM of cypripedin, respectively, compared to untreated control cells, and the viability gradually decreased to 0.70- and 0.52-fold at 72 h, whereas the lower doses (≤20 µM) of cypridedin showed no significant change over the incubation times (Fig. 1B). The apoptosis assay also confirmed the above finding; following cypripedin treatment at the concentrations of 50 and 100 µM for 72 h, the number of apoptotic nuclei were noticeably increased by 15.28% and 38.29%, respectively (Fig. 1C,D). The non-toxic doses of cypripedin were then used in the following experiments.

### Cypripedin suppresses cell migration and an anchorage-independent growth

**Figure 2:**
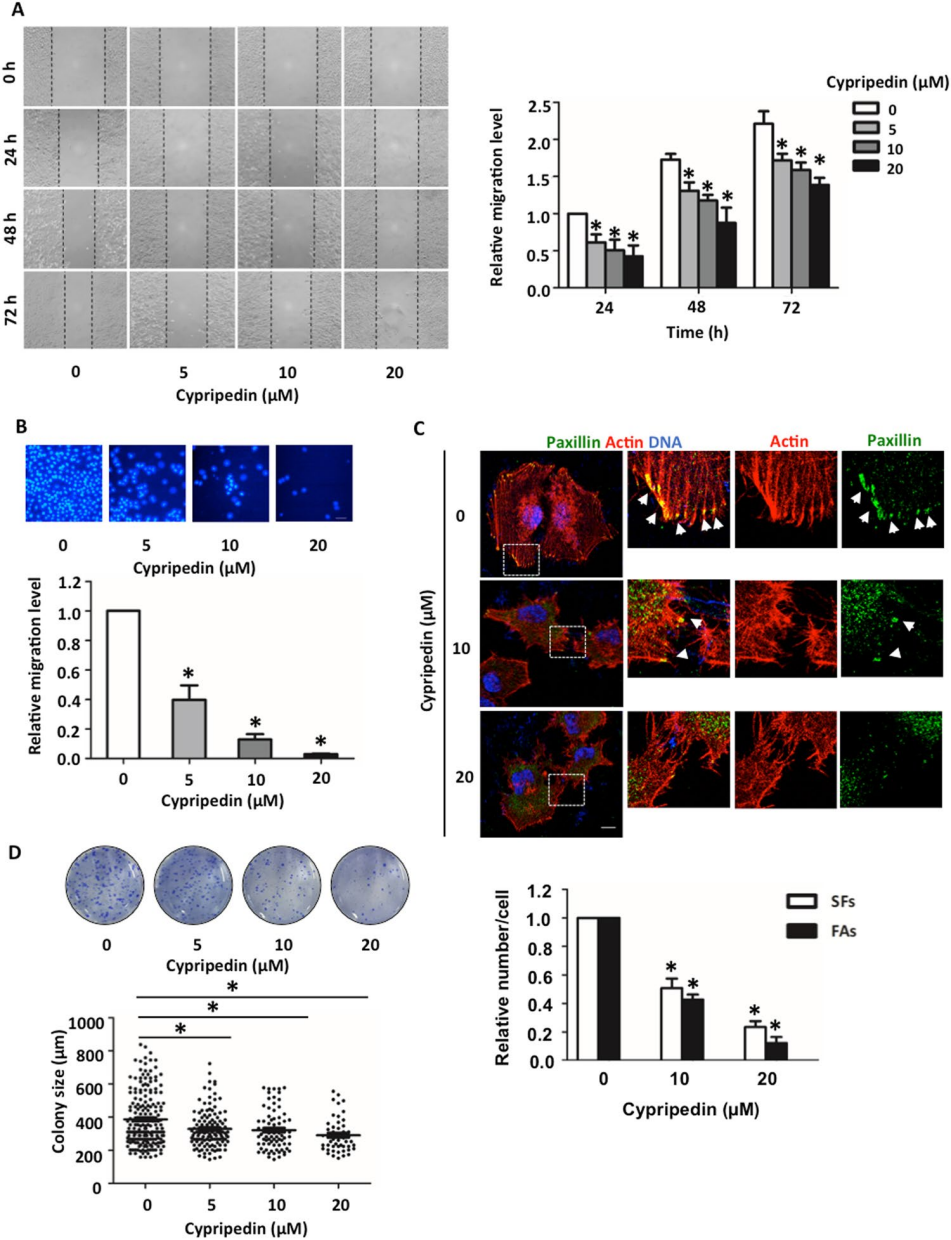
Cypripedin suppresses cell migration and an anchorage-independent growth. (**A**) H460 cells were pre-treated with non-toxic concentrations (0–20 µM) of cypripedin for 72 h, and the wound healing assay was performed. The wound space was captured and measured at 0, 24, 48 and 72 h. The wound area was calculated and presented as a relative value to the area at the initial time point. The data are presented as mean ± SEM (n = 4). *p < 0.05 compared with control cells. (**B**) The cells were treated similarly with cypripedin and were subjected to the transwell migration assay. After 18 h, the migrated cells were fixed with MeOH and were stained with DAPI. The migrated cells that were underneath of membrane were imaged by fluorescence microscopy (scale bar is 10 µm) and were calculated as the relative number of migrated cells of the cypripedin treated group over the untreated control group. The data are presented mean ± SEM (n = 4). *p < 0.05 compared with control cells. (**C**) H460 cells were seeded on cover slips and treated with non-toxic concentrations (0–20 µM) of cypripedin for 72 h. The actin stress fibres (red), focal adhesion protein paxillin (green) and nuclei staining DAPI (blue) were analysed by immunofluorescence assay and were imaged by a confocal fluorescence microscope (scale bar is 10 µm). The number of actin stress fiber (SFs) and paxllin-adhered stress fiber (FAs; arrow) were quantified. The data are presented as mean ± SEM from at least 50 cells. *p < 0.05 compared with control cells. (**D**) H460 cells were treated similarly with cypripedin (0–20 µM) for 72 h and were subjected to an anchorage-independent growth assay. After 14 d, the colonies were stained by crystal violet. The dot plot represents the value of a single colony. The data are presented as mean ± SEM (n = 4). *p < 0.05 compared with control cells.

Cancer metastasis is one of the common cancer hallmarks, and the epithelial to mesenchymal phenotype is an essential process to achieve metastasis^3^. The transformation to mesenchymal polarity was required for motile activity and for cell survival mechanisms in the detached environment that occurs during systemic circulation. To explore whether cypripedin could suppress these aggressive behaviours, cell migration and anchorage-independent growth were evaluated. The wound healing assay showed that cypripedin was able to significantly attenuate H460 cell movement in a dose-dependent manner over the experimental period (Fig. 2A). Consistently, the transwell migration assay, in which the cells vertically migrate through the porous membrane, showed that the number of cells that migrated were clearly decreased to 0.39-, 0.15- and 0.03-fold in response to 5, 10 and 20 µM of cypripedin, respectively (Fig. 2B). Since the reduction in the wound area and the number of migrating cells might be influenced by the effect of the compound on cell growth, a cell proliferation assay was conducted. Figure 1E shows that cypripedin neither enhanced nor retarded cell growth at any of the time points, indicating that the minimized locomotion of the cells resulted from effects on mobility and not on cellular growth.

Actin stress fibres and focal adhesion play an important role in providing the pooling force and adherence apparatus for cell movement to new locations^22,23^. Immunofluorescence showed that control cells displayed the dense bundle of actin (red) with an abundance of paxillin (green), a focal adhesion marker co-localized with the actin tip, and this phenotype was obviously decreased in the presence of cypripedin (Fig. 2C). These data indicated that cypripedin not only restricted cell motility but also altered the cytoskeleton organization, shifting it towards immobility.

Next, we investigated whether cypripedin was able to attenuate cell growth under detachment conditions. Since the survival mechanism of tumour cells following adherence loss is triggered by the dissociation of adherence proteins from the basement membrane, which is one of the mesenchymal phenotypes, the colony formation assay was performed. The results showed that cypripedin strongly decreased both the number and size of colonies formed (Fig. 2D). Each dot represented a single colony, the number and size of which was significantly reduced in response to cypripedin treatment compared to the control cells. These results indicated the potential effect of cypripedin on the suppression of mesenchymal behaviours.

**Figure 3:**
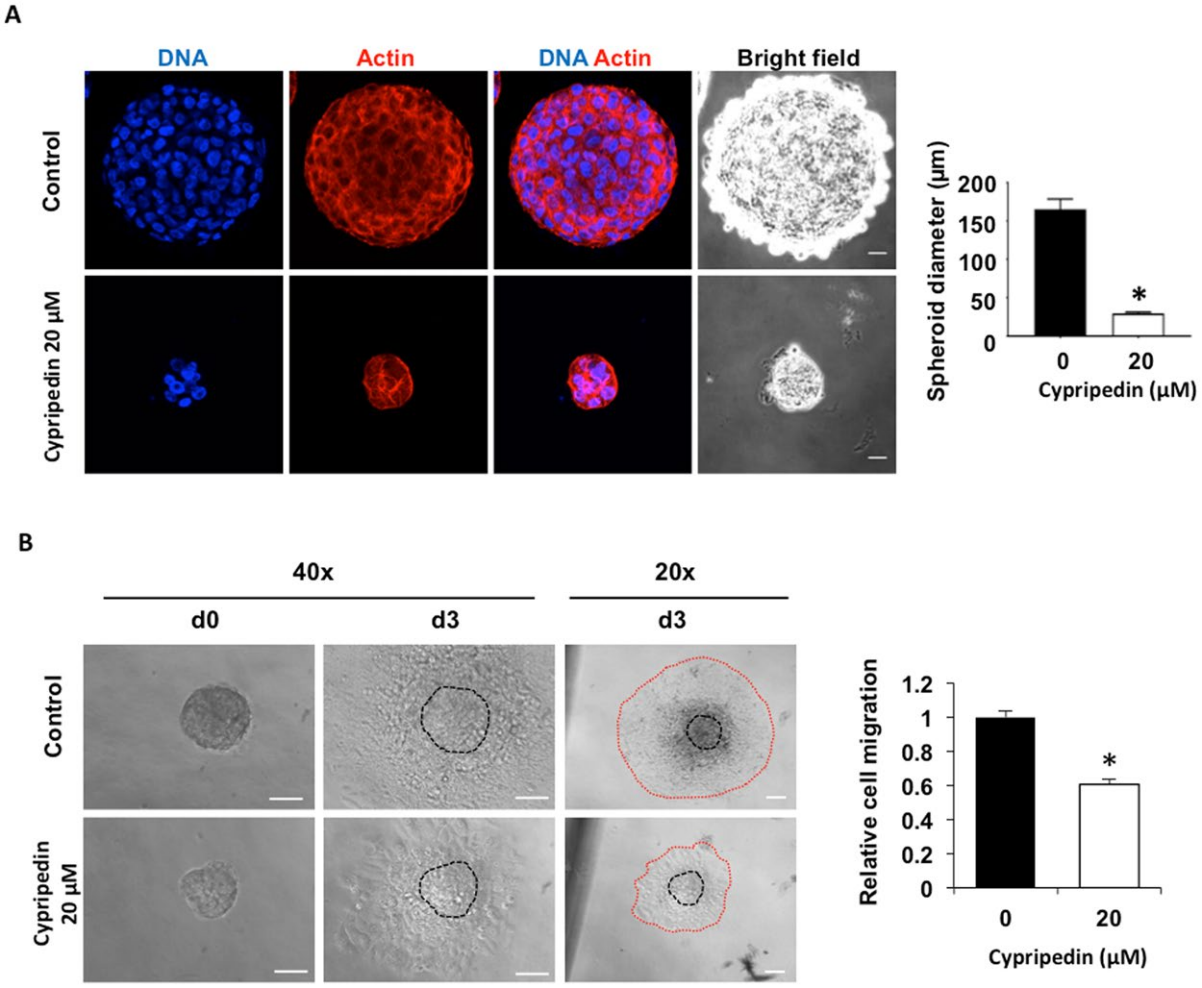
Cypripedin attenuated *in vitro* tumourigenesis and spheroid-based cell migration. (**A**) H460 cells were mixed with 4% Matrigel and cultured onto Matrigel coated-cell culture plate in the presence or absence of cypripedin (20 µM). After 10 d, spheroid was immunostained for actin (red) and DNA (blue). The data are presented as a mean of spheroid diameter ± SEM (n = 25). *p < 0.05 compared with control cells. Scale bar is 20 µm. (**B**) Spheroids were generated under detached condition as described in Method, seeded onto cell culture plate and treated with or without cypripedin (20 µM). Images were captured at d0 and d3 with 20x and 40x magnification, and cell migration was analyzed from the migrating distance (between red line and black line). The data are presented as a mean of migrating distance ± SEM (n = 15). *p < 0.05 compared with control cells. Scale bar is 100 µm.

We further extend the observation of this compound on lung tumourigenesis. The *in vitro* three-dimension tumourigenesis model provided an adequate cancer microenvironment, in which the cancer spheroid exhibits ultimately functional of the cells in metastatic context^24–27^. Cells were grown on matrix-like substance proximately to an *in vivo* condition, which pathogenically relevant to cancer progression and metastasis, in the presence or absence of cypripedin. Our data revealed that cypripedin strongly suppressed spheroidal growth (Fig. 3A). In addition, cancer cell migration from spheroid outgrowth, reflecting an *in vivo* cancer cell motility, was attenuated following cypripedin treatment (Fig. 3B). These data support the profound effect of this compound against cancer.

### Cypripedin down-regulates epithelial to mesenchymal transition markers in lung cancer H460 cells

**Figure 4:**
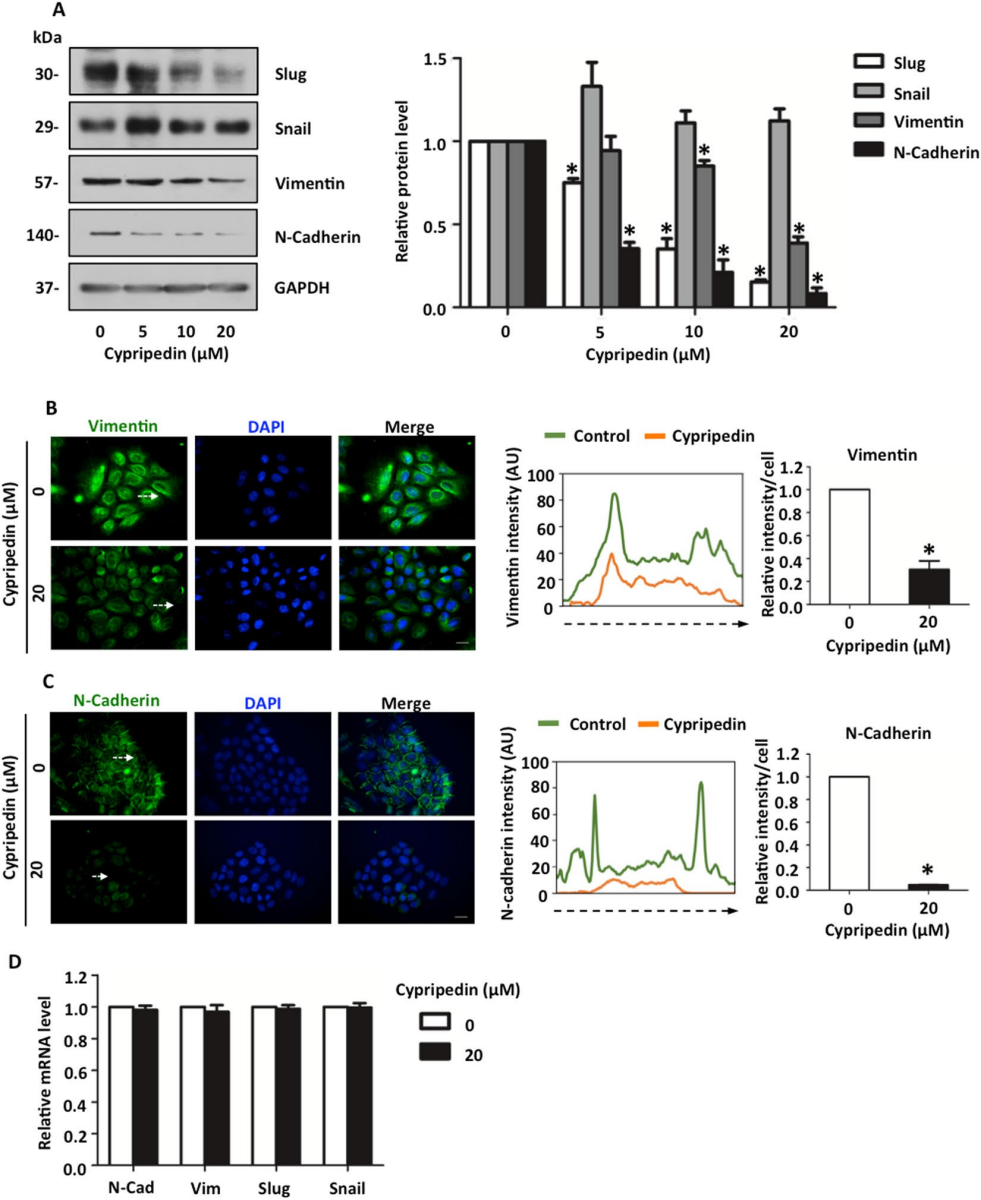
Cypripedin inhibits the epithelial to mesenchymal transition (EMT) in lung cancer H460 cells. (**A**) After H460 cells were treated with non-toxic concentration (0–20 µM) of cypripedin for 72 h, the protein expression levels of EMT markers Slug, Snail, Vimentin and N-Cadherin were analysed by Western blotting, and the intensity was qualified by densitometry. GAPDH was reprobed to confirm equal loading. The data are presented as mean ± SEM (n = 4). *p < 0.05 compared with control cells. Vimentin (**B**) and N-Cadherin (**C**) were visualized by immunofluorescence staining assay. The fluorescence intensity was analysed by ImageJ software. The diagram illustrates signal intensity along the dotted line, whereas each bar presented the relative mean intensity/cell from at least 50 cells. (**D**) The mRNA expression levels of the EMT markers were measured by quantitative RT-PCR and are presented as relative value to the untreated control cells. The data are presented as mean ± SEM (n = 4). *p < 0.05 compared with control cells.

To confirm the above observation, the key EMT regulatory proteins and their correspondence to mesenchymal characteristics were evaluated. Western blot analysis revealed that the protein levels of mesenchymal markers Slug, N-Cad and Vimentin were notably down-regulated in a dose-dependent manner (Fig. 4A). However, we failed to detect any change in Snail expression, indicating that Snail might be not a target of cypripedin. In addition, immunofluorescence assay demonstrated that an intermediate filament Vimentin, mainly localized in the cytoplasm, was extensively diminished following cypripedin treatment (Fig. 4B). We also found that N-Cad, which was enriched at the cell-cell adhesion of cells with a mesenchymal-like phenotype, was clearly reduced in the presence of such cypripedin (Fig. 4C). This cell type, which is derived from the metastasis cancer stage, exhibited a certain level of mesenchymal characteristics; although cypripedin could down-regulate N-Cad in this cell, this treatment was not able to reverse E-Cad expression (data not shown), which may be due to the entire nature of the cells. We further analysed the mRNA level of these EMT markers, and we found that there was no change in mRNA expression (Fig. 4D), indicating that the negative regulation of cypripedin on mesenchymal-related proteins might be through post-transcriptional modifications.

### Cypripedin negatively regulated epithelial to mesenchymal transition via an Akt-dependent mechanism

**Figure 5:**
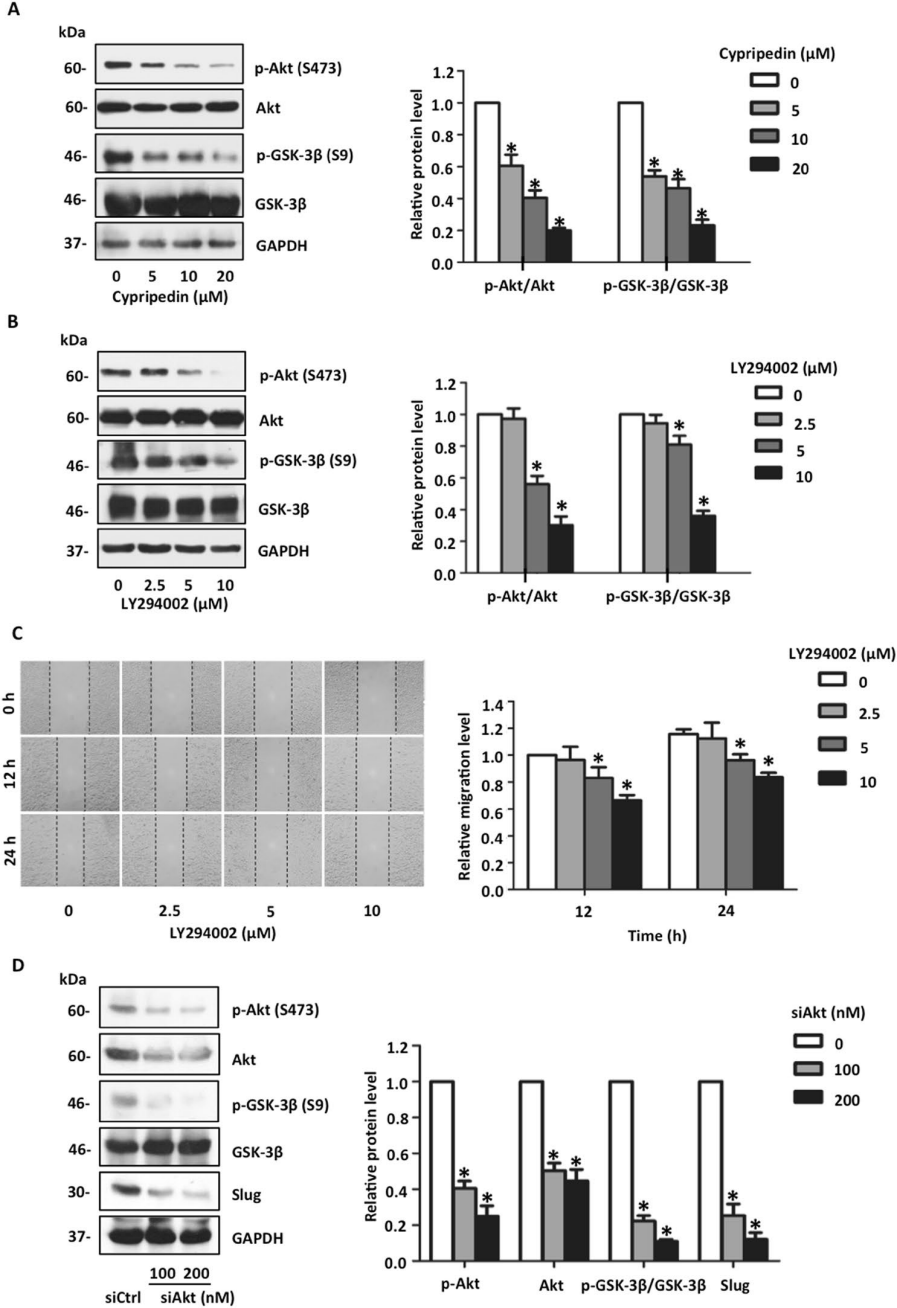
Cypripedin down-regulates Slug via an Akt-GSK-3β-dependent mechanism. (**A**) After treatment with non-toxic concentrations (0–20 µM) of cypripedin for 72 h, the protein expression levels of p-Akt (Ser 473), Akt, p-GSK-3β (Ser 9), and GSK-3β were analysed by Western blotting, and the intensity was qualified by densitometry. GAPDH was reprobed to confirm equal loading. The data are presented as mean ± SEM (n = 4). *p < 0.05 compared with control cells. (**B**) H460 cells were treated with an Akt inhibitor LY294002 (0–10 µM) for 18 h; the protein expression levels of p-Akt (Ser 473), Akt, p-GSK-3β (Ser 9) and GSK-3β were analysed by Western blotting, and the intensity was qualified by densitometry. GAPDH was reprobed to confirm equal loading. The data are presented as mean ± SEM (n = 4). *p < 0.05 compared with control cells. (**C**) After the cells were treated similarly with an Akt inhibitor LY294002 (0–10 µM), cell migration was evaluated by the wound healing assay. Wound space was captured and measured at 0, 12 and 24 h. The wound area was calculated and presented as relative value to those at initial time point. The data are presented as mean ± SEM (n = 4). *p < 0.05 compared with control cells. (**D**) H460 cells were transfected with siAkt (100 and 200 nM) or si-mismatch control. After post-transfection for 72 h, the protein expression levels of p-Akt (Ser 473), Akt, p-GSK-3β (Ser 9), GSK-3β and Slug were analysed by Western blotting, and the intensity was qualified by densitometry. GAPDH was reprobed to confirm equal loading. The data are presented as mean ± SEM (n = 4). *p < 0.05 compared with untreated control cells.

Akt pathway was widely acceptable as an enhancer of several cancer behaviours, including cancer metastasis^28^. Furthermore, the Akt signalling was required for this mesenchymal transformation through both transcriptional and post-transcriptional regulation of target proteins, especially Slug^20,29^. Next, we examined whether the Akt pathway is a target of the cypripedin-mediated suppression of the EMT process. Western blot analysis demonstrated that Akt phosphorylation, an active state of Akt, was strikingly decreased in response to cypripedin treatment, meanwhile total Akt was unchanged (Fig. 5A). Because glycogen synthase kinase-3β (GSK-3β) was responsible for Slug down-regulation via degradation mechanism, and in an exertion of Slug level, GSK-3β was required to be inactivately phosphorylated by Akt^20^. Supporting our hypothesis, the ratio of inactive GSK-3β phosphorylation to active GSK-3β was clearly diminished in the presence of cypripedin (Fig. 5A). Furthermore, the administration of TDZD-8, a selective GSK-3β inhibitor^30,31^, was able to rescue the reduction of slug mediated by such compound (Fig. S1), indicating that a decreasing in Slug level might be a result of cypripedin-restoring GSK-3β function.

To verify this finding, we treated the cells with a well-known phosphatidylinositol 3-kinase (PI3K) inhibitor LY294002 that is required for Akt activation, and we observed the effects. Figure 5B,C showed that Akt phosphorylation was significantly decreased after exposure to LY294002, GSK-3β phosphorylation was reduced, and cell motility was suppressed. In addition, we transfected the cells with specific small interference mRNA targeting Akt. Western blot analysis revealed that the expression of Akt and its active form were obviously decreased in response to this manipulation, and the inactive state of GSK-3β was notably attenuated (Fig. 5D). Interestingly, the Slug level was significantly reduced, similar to the effects of cypripedin treatment. In the other hand, constitutive Akt overexpression could rescue the inhibitory effect of this treatment on cell migration and Slug expression (Fig. S2). These data support the hypothesis that cypripedin inhibits the mesenchymal transformation process in a mechanism involving the Akt-GSK3β-Slug axis.

### Cypripedin enhances Slug degradation via ubiquitin-proteasomal mechanism

**Figure 6:**
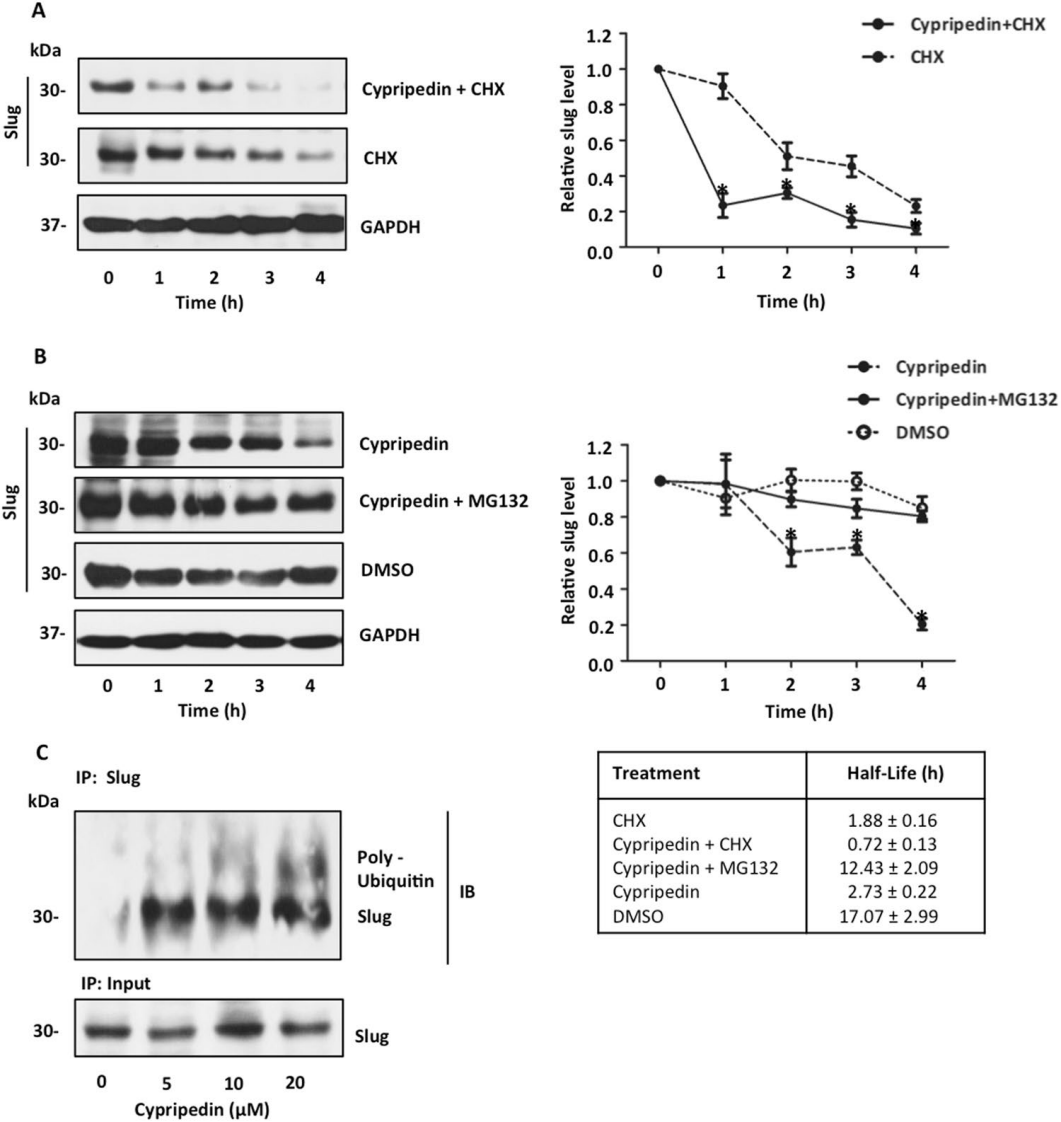
Cypripedin mediates Slug degradation via ubiquitin-proteasomal pathway. (**A**) H460 cells were pre-treated with cycloheximide (CHX, 10 µg/mL) for 1 h before incubation with or without 20 µM of cypripedin for 0–4 h. (**B**) H460 cells were pre-treated with or without a proteasome inhibitor, MG132 (10 µM) for 1 h, before treatment with 20 µM of cypripedin for 0–4 h. Slug expression was evaluated by Western blot analysis, and the intensity was qualified by densitometry. GAPDH was reprobed to confirm equal loading. The relative Slug level over the experiment periods was presented, and the half-life was calculated. The data are presented as mean ± SEM (n = 4). *p < 0.05 compared with control cells. (**C**) H460 cells were pre-treated with MG132 10 µM for 1 h, followed by incubation with cypripedin (0–20 µM) for 3 h. The Slug ubiquitination was analysed by immunoprecipitation assay. The lysates were obtained, and then Slug was pulled down with anti-Slug antibody, and the Slug pull-down samples were collected and subjected to immunoblotting to confirm the equal levels of Slug substrate.

The degradation of Slug by the proteasomal pathway determined the Slug level, and hence, its function, and our finding suggested that cypripedin might affect this post-transcriptional modification. To verify this hypothesis, first we treated the cells with a protein synthesis inhibitor cycloheximide (CHX) in the presence or absence of cypripedin (20 μM), and the degradation rate was evaluated. The results showed that Slug levels were gradually reduced in accordance with CHX treatment, and the half-life of Slug was approximately 1.88 ± 0.16 h (Fig. 6A). Interestingly, combination cypripedin and CHX treatment accelerated the Slug degradation rate, and its half-life was reduced to 0.72 ± 0.13 h. Second, we incubated the cells with cypripedin and a proteasomal inhibitor MG132, and we examined its preventive effects. A single treatment of cypripedin caused a substantial decrease in Slug levels, with a half-life of 2.73 ± 0.22 h. The rate of Slug degradation declined following MG132 treatment, and the half-life of Slug was extended to approximately 12 h (Fig. 6B), suggesting that cypripedin mediated Slug elimination rather than modulated its synthesis.

Since ubiquitination is a required process for targeting proteins for proteasome-mediated degradation^32,33^, to strengthen our observation, we examined Slug poly-ubiquitination by immunoprecipitation assay. We pulled down Slug with a specific antibody and immunoblotted with a ubiquitin-specific antibody, and we found that the levels of poly-ubiquitinated Slug appeared to increase in response to cypripedin treatment (Fig. 6C). These results indicated that cypripedin enhanced Slug elimination via a ubiquitin-proteasomal dependent mechanism.

### Cypripedin attenuates epithelial to mesenchymal transition in lung cancer H23 cells

**Figure 7:**
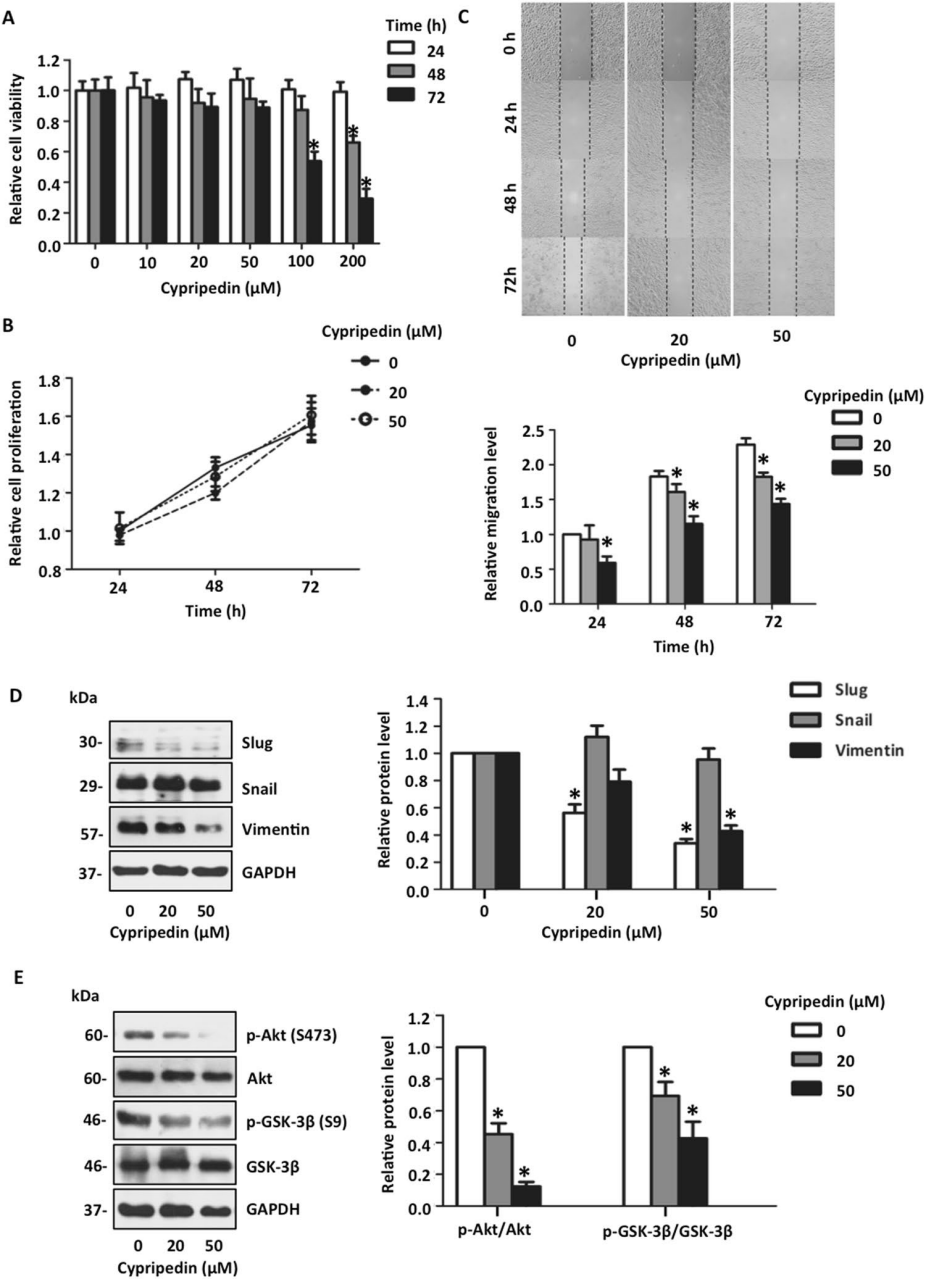
Cypripedin suppresses the epithelial to mesenchymal transition (EMT) in lung cancer H23 cells. (**A**) H23 cells were treated with various concentrations (0–200 µM) of cypripedin for 24, 48 and 72 h and cell viability was measured by MTT assay. The data are presented as mean ± SEM (n = 4). *p < 0.05 compared with control cells. (**B**) H23 cells were treated with non-toxic concentrations (0–50 µM) of cypripedin for 24, 48 and 72 h, and cell growth was examined by cell proliferation assay and presented as a relative value. The data are presented as mean ± SEM (n = 4). *p < 0.05 compared with control cells. (**C**) H23 cells were pre-treated with non-toxic concentrations (0–50 µM) of cypripedin for 72 h, and the wound healing assay was performed. Wound space was captured and measured at 0, 24, 48 and 72 h. The wound area was calculated and presented as a relative value to those at initial time points. The data are presented as mean ± SEM (n = 4). *p < 0.05 compared with control cells. (**D**) After H23 cells were treated with non-toxic concentrations (0–50 µM) of cypripedin for 72 h, the protein expression levels of EMT markers Slug, Snail and Vimentin were analysed by Western blotting, and the intensity was qualified by densitometry. GAPDH was reprobed to confirm equal loading. The data are presented as mean ± SEM (n = 4). *p < 0.05 compared with control cells. (**E**) The effect of cypripedin on EMT-regulated proteins was investigated using Western blot analysis. After treatment with non-toxic concentrations (0–50 µM) of cypripedin for 72 h, the protein expression levels of p-Akt (Ser 473), Akt, p-GSK-3β (Ser 9), GSK-3β and Slug were analysed by Western blotting, and the intensity was qualified by densitometry. GAPDH was reprobed to confirm equal loading. The data are presented as mean ± SEM (n = 4). *p < 0.05 compared with control cells.

To provide supportive evidence for the potential effect of cypripedin in lung cancer, the suppressive effect of this treatment was also evaluated in another lung cancer type, H23 cells. First, the cytotoxic and cell proliferative effects of cypripedin on H23 cells was examined, and we found that neither toxicity nor cell growth suppression was detectable in the cypripedin-treated group over the same dose range (Fig. 7A,B). Second, the cell motility in response to cypripedin was investigated by a wound healing assay. Figure 7C demonstrated that the wound area that remained was slightly diminished in the presence of such treatment, while the wound space became remarkably narrow in the control group. Next, we evaluated the underlying mechanism of cypripedin on the EMT process; the expression of markers related to EMT were analysed. Western blot analysis revealed that Slug and Vimentin were clearly down-regulated, whereas Snail remained unchanged (Fig. 7D). Cypripedin suppressed Akt activation, and thus, reduced the inactive form of GSK-3β phosphorylation accordingly (Fig. 7E). These data supported our hypothesis that cypripedin suppresses lung cancer mesenchyme-like phenotypes and that the underlying mechanism involves the inhibition of Akt that leads to the stimulation of GSK-3β-mediated Slug degradation.

## Discussion

**Figure 8:**
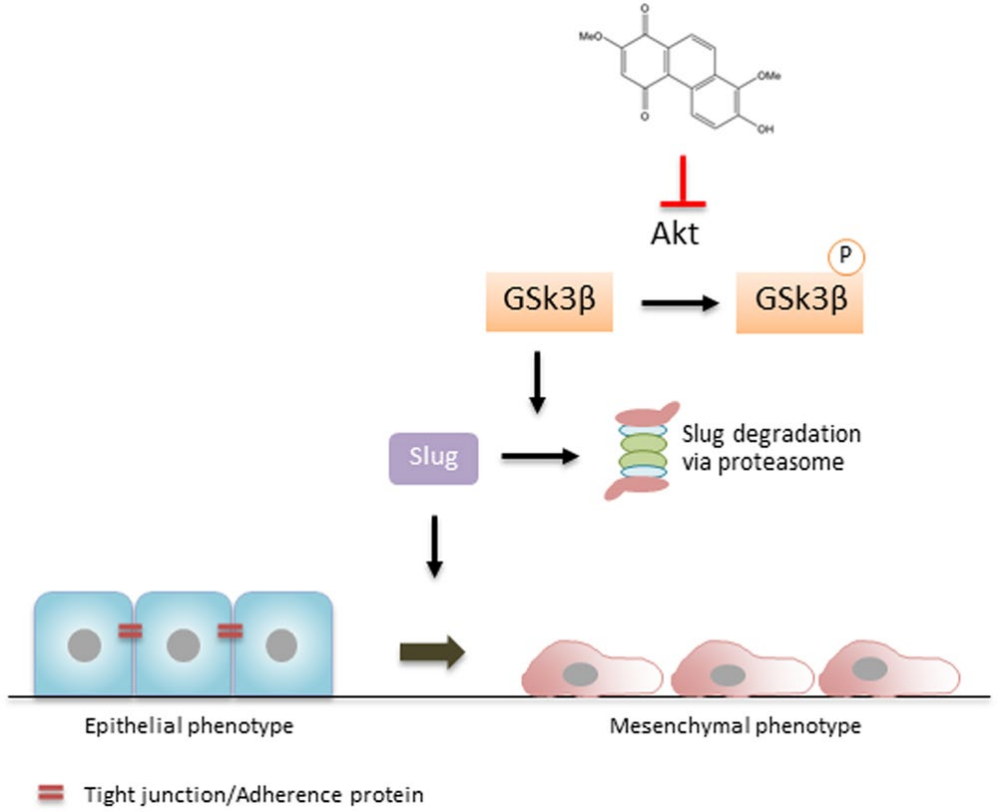
A schematic diagram summarizes the underlying mechanism of cypripedin-suppressing EMT in lung cancer cells.

In the present study, we demonstrated the anti-metastasis potential of cypripedin on lung cancer cells using H460 and H23 cells as an *in vitro* model. Cypripedin was able to suppress the transition from epithelial to mesenchymal phenotypes, both the migratory behaviour and colony formation under detached cellular conditions were remarkably decreased, along with the attenuation of *in vitro* tumourigenesis and spheroid-based cell migration. The mesenchymal protein markers Slug, Vimentin and N-Cad were obviously down-regulated with cypripedin treatment. Notably, the negative regulation of cypripedin on this transformation process was caused by the attenuation of Akt activity. Using a chemical inhibitor and genetic manipulation targeting Akt function and activity, we found that the Akt-regulated suppression of GSK-3β activity was reversed, similar to those observations in cypripedin treatment. In addition, Slug appeared to be reduced as a consequence of GSK-3β stimulation, which is responsible for Slug degradation via a proteasomal mechanism (Fig. 8).

Previous studies have reported the attractive anti-cancer effects of phenolic compounds from Thai orchids, *Dendrobium densiflorum*. Moscatilin exhibited an inhibitory effect on cell motility and invasion in a focal adhesion kinase-dependent manner^18^. Gigantol displayed anti-metastasis action by sensitization of detachment-induced apoptosis and by impeding cell migration in an involvement with the EMT mechanism^19,34^. Since substances with quinone derivatives have demonstrated a strong anti-cancer activity, including apototsis induction^35,36^, our finding also provided an additional pharmacological activity of a phenanthrenequinone compound from this plant as a potent suppressor of the EMT transformation in lung cancer cells.

It is noteworthy that EMT served as an essential mechanism behind the morphologic and genetic alteration, driving the movability and survival of the metastatic cancer cells^3^. The conversion of cells from the epithelial to mesenchymal phenotypes is characterized by the deconstruction of tight junction proteins, favouring cellular detachment and the establishment of new front-rear polarization and is associated with Cadherin switching^4,6^. The altered translocation or expression pattern of these transmembrane components initiated aberrant differentiation of the cells, contributing to carcinogenesis behaviour^11^. Many studies have indicated that N-Cad is an interesting target for lung cancer treatment^37^. Our finding also revealed that cypripedin interfered with the Cadherin swithching, causing a reduction in N-Cad (Fig. 4).

Emerging evidence indicates that the regulation of Cadherin switching could occur through the transcriptional and post-transcriptional modification^38–40^. Although several transcription factors, including Snail and Slug, regulate the expression of Cadherin subtypes^41^, recent clinical data suggested that the up-regulation of N-Cad is significantly related to the increased level of Slug, independently to Snail^42^, and Slug has become emphasized as a dominant oncogenic factor in lung cancer pathogenesis^43^. Slug was a target of cypripedin-mediated suppression of EMT in this cancer. However, our data demonstrated that the mRNA level of N-Cad was unchanged. Since the translation of N-Cad has been shown to be regulated by the PI3K/mTOR pathways^44^ and multiple types of micro RNAs^40,45,46^, it might be possible that cypripedin influenced N-Cad post-transcription, in addition to its synthesis, via these regulatory factors and might be irrelevant to the regulation by Slug. Similarly, our data also showed that Vimentin mRNA level was not altered in response to cypripedin, although Slug has been reported as a Vimentin transcriptional regulation^47^ (Fig. 4). It might be possible that various combinative factors participated in Vimentin synthesis including AP1^48^, ZBP-89/Sp1^49^ and diverse microRNAs^50,51^, which were not affected by such treatment. Recent study also supported this hypothesis that Slug was associated with Vimentin subcellular localization, but had slightly effect on its expression^52^. Furthermore, cypripedin might interfere Vimentin protein level via Akt mechanism. It has reported that Akt plays a critical role on post-transcription of Vimentin. Vimentin phosphorylated by Akt became more longevity which prevents its caspase-mediated proteolysis^53^. Its downregulation might be, in part of, that cypripedin attenuated Akt activity-stabilizing Vimentin, rather than the direct effect of Slug on its transcriptional regulation. The precise mechanism of this compound on the post-transcriptional regulation of N-Cad and Vimentin would be worthy of further study.

According to our observation, the decreased expression of Slug, without a reduction of its mRNA levels, along with an enhanced protein degredation rate, suggested that the stability of this protein was affected greatly by cypripedin. Even though Slug is considered to have a relatively short half-life^54^, cypripedin was able to accelerate its removal rate (Fig. 6). It is widely accepted that the Akt pathway participates in various biological responses associated with survival, proliferation and migration of cancer cells that are related to the conduction of EMT though particular signals^28^. Accumulated evidence indicates that Slug is involved in an Akt-mediated transition to the mechanchymal-like phenotype, in which Akt inhibits the degredation of Slug^20,55^. Under this signal transduction pathway, GSK-3β, which was required for Slug instability, was repressed via phosphorylation at Ser 9 by Akt^56^. We found that cypripedin clearly suppressed the Akt signalling pathway, in turn rescuing GSK-3β function and, consequently, Slug protein destabilization.

Previous studies indicated that the stability of Slug was extended in basal-like breast cancer by abolishing its proteasomal degradation^54,57^, and the enhancement of proteasomal function led to the down-regulation of this protein^20,55^. It is of note that ubiquitination plays an important role on the post-translational modification process that involves proteasome-mediated protein elimination. Protein targets are marked by covalent attachment of the small protein ubiquitin prior to recognition by the proteasome, where the catalytic activity of protein degradation occurs^32^. The existence and function of several proteins, including Slug, are determined by this degradation pathway^54,58^, supporting to our finding that cypripedin mediated the down-regulation of Slug through the proteasomal degradation mechanism.

Although Slug and Snail are members of Snail family transcription factor and share similarly degradation pathway via GSK-3β^59,60^, cypripedin-suppressing Akt/GSK-3β could not mediate Snail reduction. Since their structural homologs are slightly different, they might have diverse binding partners including the stabilize-regulatory proteins. Accumulative studies reported that the existence of Snail was controlled by various cues independently of Akt/GSK-3β. The phosphorylation at serine 11 on Snail by protein kinase D1 (PKD1) was required for FOXB11-mediated degradation^61,62^. The dephosphorylation of Snail by the small C-terminal domain (CTS) phosphatases (SCPs) was able to prevent Snail destabilization^63^. In addition, TNFα stabilized Snail via COP9 signalosome 2 (CSN2)- pathway^64^, and HSP27 was shown to interact with and protect Snail from its proteasomal deprivation^65^. Our data suggests that Snail degradation might be a dependency of other mechanisms that less influenced by cypripedin.

Taken together, these results demonstrated that cypripedin suppresses the mesenchymal transition in lung cancer via inhibition of Akt, which in turn activates GSK-3β and leads to Slug degradation. This new discovery on the pharmacological effect of cypripedin could support future research and development of cypripedin as an alternative therapy for lung cancer patients.

## Materials and Methods

### Cell culture and reagents

Human lung cancer H460 and H23 cells were obtained from American Type Culture Collection (ATCC) (Manassas, VA, USA) and were cultured in Roswell Park Memorial Institute (RPMI)-1640 medium containing 10% fetal bovine serum (FBS), 2 mM L-glutamine, 100 units/mL streptomycin and penicillin at 37 °C in a humidified atmosphere containing 5% CO_2_. RPMI-1640 medium, L-glutamine, penicillin, streptomycin, FBS, 0.25% trypsin-EDTA, and phosphate-buffered saline (PBS) were purchased from GIBCO (Grand Island, NY, USA). 3-(4,5-dimethylthiazol-2-yl)-2,5-diphenyltetrazolium bromide (MTT) was purchased from Invitrogen (Carlsbad, CA, USA). DMSO, Hoechst 33342, 4′,6-diamidino-2-phenylindole dihydrochloride (DAPI), MG132, TDZD-8, cycloheximide (CHX) and chloroform were obtained from Sigma (St. Louis, MO, USA). Agar noble was purchased from BD Biosciences (Franklin Lakes, NJ, USA).

Primary antibodies were used as follows: mouse anti-paxillin (BD Biosciences, Franklin Lakes, NJ, USA); mouse anti-GAPDH (CST, Beverly, MA, USA); rabbit anti-Slug (CST, Beverly, MA, USA); rabbit anti-Vimentin (CST, Beverly, MA, USA); rabbit anti-N-Cadherin (CST, Beverly, MA, USA); rabbit anti-phosphorylated Akt (S473; CST, Beverly, MA, USA); rabbit anti-Akt (CST, Beverly, MA, USA); rabbit anti-phosphorylated GSK-3β (Ser9; CST, Beverly, MA, USA) and rabbit anti-GSK-3β (CST, Beverly, MA, USA). Secondary antibodies used in this study were anti-rabbit IgG HRP-linked (CST, Beverly, MA, USA); anti-mouse IgG HRP-linked (CST, Beverly, MA, USA); Alexa Fluor 488-conjugated goat anti-rabbit IgG (Life Technologies Eugene, OR, USA); Alexa Fluor 488-conjugated goat anti-mouse IgG (Life Technologies Eugene, OR, USA) and Alexa Fluor 568 phalloidin (Life Technologies Eugene, OR, USA).

### Cypripedin preparation

Cypripedin was isolated from *Dendrobium densiflorum* using methanol extraction and purified by column chromatography (C-18, H_2_O-MeOH, gradient). The structure of cypripedin was determined through analysis of NMR (supplementary information), and its purity was evaluated by HPLC and NMR which cypripedin with more than 95% purity was used in this study. The chemical structure was illustrated in Fig. 1A. For cypripedin preparation in the experiments, it was dissolved in dimethylsulfoxide (DMSO) as a stock solution, which was further diluted with cell culture media to the desired working concentrations. The final concentration of DMSO that was used in all experiments was less than 0.1%, which showed no cytotoxicity. The control cells that were exposed to equal concentrations of DMSO were employed for comparison to the effect of the cypripedin-treated group.

### Cytotoxic and cell proliferative assay

For cytotoxic testing, the cells were seeded at a density of 10,000 cells/well in 96-well plates and incubated at 37 °C overnight for cell attachment. After the cells were treated with various concentrations (0–100 µM) of cypripedin for 24, 48 and 72 h, 10 µL of MTT solution (5 mg/mL) was added and incubated at 37 °C for 4 h. The formazan crystals were dissolved with the addition of 100 µL of DMSO. The intensity of formazan product was detected at an absorbance wavelength of 570 nm with a microplate reader (Perkin Elmer VICTOR^3^/Wallac 1420). The cell viability was calculated as follows: relative cell viability = optical density of treated group/optical density of control group.

For the assessment of cell proliferation, the cells were pre-treated with various non-toxic concentrations of cypripedin (0–20 µM) for 72 h, plated onto 96-well plates at 3,000 cells/well, and incubated at 37 °C for 24, 48 and 72 h. After the incubation period, 10 µL of MTT solution (5 mg/mL) was added to each well and was incubated at 37 °C for 4 h. The formazan crystals were dissolved with 100 µL of DMSO, and the intensity of soluble formazan was detected at an absorbance wavelength of 570 nm by a microplate reader. The cell proliferation was calculated as value relative to the control group at each time point as follows: relative cell proliferation = optical density of treated group/ optical density of control group.

### Apoptosis assay

Apoptotic cells were determined using a fluorescent nuclear staining dye Hoechst 33342; 8,000 cells/well were seeded onto 96-well plates and incubated at 37 °C overnight. The cells were treated with cypripedin (0–100 µM) for 72 h, followed by incubation with Hoechst 33342 (10 µg/mL) for 30 min in the dark. Nuclear condensation and DNA fragmentation of apoptotic cells were visualized and scored from six random fields using fluorescence microscopy (Nikon Inverted Microscope Eclipse Ti-U Ti-U/B, NY, USA). The data were calculated and were presented as a percentage of apoptotic cells.

### Anchorage-independent growth assay

Cells were pre-treated with cypripedin (0–20 µM) for 72 h and were then subjected to a colony formation assay. The bottom layer of soft agar was prepared by the addition of 0.5% agarose in cell culture media on a 24-well plate. After the agarose solidified, an upper cellular layer containing suspended cells at a density of 1,000 cells/well and 0.3% agarose in media was poured onto the bottom layer and was incubated for 14 d. The cell culture media was replaced every 3 d to prevent the agar from drying. Colonies were stained with 0.01% crystal violet at room temperature for 30 min and washed five times with deionized water. The number of and size of the colonies were visualized and analysed by ImageJ software using a particle analysis plugin.

### Cell migration assay

The motility of the cells was evaluated by wound healing and transwell migration assays. For wound healing assays, the cells were pre-treated with cypripedin (0–20 µM) for 72 h and then plated onto 24-well plates at a density of 1.5 × 10^5^ cells/well. After the cells reached confluence, a 10-µL pipette tip was used to generate a wound space, and the cell debris was removed by washing the cells with PBS. The media was replaced with RPMI containing 1% FBS. After that, photographs of the wounds were captured at indicated time points by inverted microscope.

For the transwell migration assay, 5 × 10^4^ cells that were pre-treated with cypripedin were plated onto the upper chamber of 24-well transwell plates containing RPMI with 1% FBS, and 500 µL of complete media was added into the lower chamber. The cells were allowed to migrate to the lower chamber for 18 h at 37 °C. The non-migrated cells on the upper chamber were removed by cotton-swab, and the cells underneath of membrane were fixed with cold ethanol and stained with DAPI in the dark. The cells from six random fields were then photographed under a fluorescence microscope (Nikon Inverted Microscope Eclipse Ti-U Ti-U/B, NY, USA). The number of migrated cells was calculated and represented as a relative value to the number of migrated cells in the control groups.

### RNA isolation, reverse transcription and quantitative real-time PCR (qRT-PCR)

Cells were treated with cypripedin for 72 h, and total RNA was isolated using GENEzol reagent (Geneaid Biotech, Shijr, New Taipei, Taiwan) and RNAprep Pure Kit (TIANGEN^®^ Biotech, Xuhui, Shanghai, China). RNA was reverse transcribed to cDNA by ProtoScript^®^ II Reverse Transcriptase (NEB^®^ England, Ipswich, MA, USA) following the manufacturer’s instructions. The qRT-PCR assay was prepared in a total volume of 10 µL reaction mixture containing 5 µL of 2× iTaq^™^ Universal SYBR^®^ Green Supermix (Bio-Rad Laboratories, Hercules, CA, USA), 0.2 µM of each primer (Table S1), and 500 ng of cDNA template. The real-time PCR assay was performed in an IO-RAD T100^™^ Thermal Cycler (Bio-Rad Laboratories, Hercules, CA, USA) with the programme for amplifying RNA as follows: 1 cycle of 95 °C for 10 min, followed by 35 cycles of 95 °C for 30 sec, and 60 °C for 30 sec. The *GAPDH* gene was used as a constitutive control. All samples were conducted in triplicate, and the data were calculated according to the ΔΔC_t_ method^66^.

### Immunofluorescence assay

Cells were plated at a density of 2,000 cells/coverslip in 24-well plates and were treated with cypripedin for 72 h. The cells were fixed with 4% paraformaldehyde for 20 min in the dark, permeabilized with 0.1% Triton-x in PBS (500 µL/well) for 10 min, and blocked with 4% BSA in PBS at room temperature for 30 min. After the cells were incubated with primary antibodies at 4 °C overnight, the cells were washed with PBS and incubated with secondary antibody at room temperature for 1 h in the dark. The coverslips were washed with PBS containing DAPI, rinsed with deionized water and mounted by FluorSave (EMD Millipore, Billerica, MA, USA). Confocal images were acquired by either Zeiss LSM880 (Carl Zeiss) through a Plan-Apochromat 63x/1.40 N.A. or by a fluorescence microscope with a 40x objective lens (Nikon Inverted Microscope Eclipse Ti-U Ti-U/B), and the analysis was performed by ImageJ software (NIH).

### *In vitro* three-dimensional tumourigenesis assay

*In vitro* tumourigenesis assay was performed as described previously with slightly modification^67,68^. Cell culture plates were coated with 0.5% Matrigel^TM^ (BD Biosciences, NJ, USA), and dry over night at 37 °C. Cell suspension containing cypripedin and 4% Martigel^TM^ were cultured on coated plate, and the culture medium were replaced every 3 d to prevent the dryness. After 10 d, spheroid was fixed with 4% paraformaldehyde for 20 min, permeabilized with 0.1% Triton-x in PBS, and incubated with phalloidin-Alexa Fluor 568 for 2 h. The spheroids were imaged by Confocal microscope (Fluoview FV10i, Olympus) and analyzed by ImageJ software.

### *In vitro* tumour spheroid-based migration assay

*In vitro* cell migration from tumour spheroid was performed as previously reported with slightly modification^69^. Tumor spheroids were generated as described above and plated on 96-well plate. After adherent, spheroids were treated with cypriperdin and images were obtained at day 0 and 3 by inverted microscope with 20x and 40x magnification. Cell migration rate was measured by ImageJ software, and analyzed from the diameter changed between time point relatively to day 0.

### Small interference RNA Transfection assay

The siRNA used in the experiments were synthesized and annealed as follows:

si-Akt, sense: 5′-GGAGAUCAUGCAGCAUCGC-3′ and anti-sense: 5′-GCGAUGCUGCAUGAUCUCC-3′: si-mismatch control, sense: 5′-GGGAAUCAUAAAGCAUUUC-3′ and anti-sense: 5′-CCGGGGCUGCAUAAACUUC-3′.

Cells (10^6^ cells/dish) were grown on a 60-mm dish overnight, and transfected with 100 and 200 nM siRNA against Akt using Lipofectamine^®^ RNAiMAX (Invitrogen, Carlsbad CA, USA), according to manufacturer’s protocol. Briefly, the siRNA was incubated with Lipofectamine^®^ RNAiMAX for 15 min in Opti-MEM media at room temperature, the mixture was then added dropwise onto the cells. After incubation for 72 h, the cells were subjected to further experiments.

### Western blot analysis

After the indicated treatment, the cells were lysed with TMEM lysis buffer containing 20 mM Tris-HCl pH 7.5, 1 mM MgCl_2_, 150 mM NaCl, 20 mM NaF, 0.5% sodium deoxychlorate, 1% nonidet-40, 0.1 mM phenylmethylsulfonyl fluoride, and protease inhibitor cocktail (Roche diagnostics, Indianapolis, IN, USA) on ice for 40 min. The supernatant was collected by centrifugation at 20,000 xg at 4 °C for 15 min. The protein content was measured by BSA protein assay kit (CST, Beverly, MA, USA). An equal amount of protein was denatured by boiling at 95 °C for 5 min with 6X sampling buffer. The proteins were then separated by SDS-PAGE and were electrotransferred to a PVDF membrane (Bio-Rad Laboratories, Hercules, CA, USA). The membrane was blocked in 5% skim milk for 1 h and was incubated with primary antibodies at 4 °C overnight. The membranes were then washed with TBS-T (1 M Tris-HCl pH 7.5, 2.5 M NaCl and 0.075% Tween 20) and were incubated with peroxidase-conjugated secondary antibodies for 2 h at room temperature. The protein expressions were visualized by enhanced chemiluminescence system using SuperSignal West Pico (Thermo Fisher Scientific, MA, USA) and Immobilon Western (EMD Millipore, Billerica, MA, USA) and were quantified using ImageJ software (NIH).

### Immunoprecipitation assay

After the indicated treatment, the cells were pre-treated with MG132 (10 µM) at 37 °C for 1 h, prior to incubation with cypripedin at 37 °C for 3 h. H460 cells were washed with cold PBS and lysed with TMEN buffer containing 1% NP-40 for 40 min on ice. The supernatant was separated by centrifugation at 20,000 xg at 4 °C for 20 min and was pre-cleared with protein G sepharose beads (GE Healthcare, Uppsala, Sweden). The supernatant was collected by centrifugation at 2,000 xg at 4 °C for 3 min and was incubated with anti-Slug antibody overnight at 4 °C. The ubiquitinated Slug was pulled down by the addition of protein G sepharose beads. The complexes were washed 5 times with TMEN buffer containing 0.5% NP. The precipitates were boiled at 96 °C for 10 min with a sample buffer and were analysed for Slug ubiquitination by immunoblotting.

### Statistical analysis

Data are presented as mean ± SD at least four-independent experiments, and all data were analyzed using Prism 7 (GraphPad Software, Inc., San Diego, CA, USA). One-way ANOVA with Tukey’s Multiple Comparison Test was applied for determination the statistical significance between control and treatment groups with *P*-values < 0.05.

## Electronic supplementary material

Supplementary infomation
