# Supplementary material for: Cypripedin diminishes an epithelial-to-mesenchymal transition in non-small cell lung cancer cells through suppression of Akt/GSK-3β signalling
Source: Sci Rep. 2018 May 22;8:8009. doi: 10.1038/s41598-018-25657-5 (PMC5964153; doi:10.1038/s41598-018-25657-5)
Supplement: Supplementary file 1 — Supplementary infomation [file 41598_2018_25657_MOESM1_ESM.docx]

**Cypripedin diminishes an epithelial-to-mesenchymal transition in non-small cell lung cancer cells through suppression of Akt/GSK-3β signalling**

Surassawadee Treesuwan, Boonchoo Sritularak, Pithi Chanvorachote, Varisa Pongrakhananon

**Supplementary methods**

*Spectral data of cypripedin*

The structure of cypripedin was determined through analysis of NMR using Bruker Avance DPX-300 FT-NMR spectrometer, and mass spectrum was recorded on a Bruker micro TOF mass spectrometer (ESI-MS) (Fig. 1A); C_16_H_12_O_5_; HR-ESI-MS [M+Na]^+^ at *m/z* 307.0582 (calcd. for 307.0582, C_16_H_12_O_5_Na); ^1^H NMR (300 MHz, acetone-*d*_6_) δ: 9.27 (1H, d, *J* = 9.6 Hz, H-5), 8.37 (1H, d, *J* = 9.0 Hz, H-9), 8.09 (1H, d, *J* = 9.0 Hz, H-10), 7.43 (1H, d, *J* = 9.6 Hz, H-6), 6.20 (1H, s, H-3), 3.94 (3H, s, MeO-8), 3.93 (3H, s, MeO-2); ^13^C NMR (75 MHz, acetone-*d*_6_) δ: 189.1 (C-4), 181.2 (C-1), 159.6 (C-2), 149.5 (C-7), 141.1 (C-8), 134.0 (C-8a), 130.0 (C-10a), 128.3 (C-4a), 127.2 (C-9), 126.2 (C-5), 125.7 (C-4b), 123.4 (C-6), 122.7 (C-10), 111.9 (C-3), 61.5 (MeO-8), 56.7 (MeO-2).

*Akt plasmid transfection*

The constitutively active Akt plasmid (Akt-WT) was kindly gift by Dr. Sudjit Luanpitpong (Siriraj Center of Excellence for Stem Cell Research, Thailand). H460 cells were transfected by using Lipofectamin^®^2000 (Invitrogen, Carlsbad CA, USA), according to manufacturer’s protocol. A 2 µg of pcDNA3-Akt or pcDNA3 control vectors were incubated with

Lipofectamin^®^2000 for 20 min, the mixture was then added dropwise onto the cells. After incubation for 24 h, the cells were subjected to further experiments.

**Table S1.** Sequences of the primers used in the experiment

| **Genes** | **Forward primer** | **Reverse primer** |
| --- | --- | --- |
| *NCAD* | 5’-GACCGAGAATCACCAAATGTG-3’ | 5’-GCGTTCCTGTTCCACTCATAG-3’ |
| *SNAIL* | 5’-CTAGCGAGTGGTTCTTCTGC-3’ | 5’-GTAGTTAGGCTTCCGATTGGG-3’ |
| *SLUG* | 5’-AGCATTTCAACGCCTCCA-3’ | 5’-GGATCTCTGGTTGTGGTATGAC-3’ |
| *VIMENTIN* | 5’-ACCCTGCAATCTTTCAGACAG-3’ | 5’-GATTCCACTTTGCGTTCAAGG-3’ |
| *GAPDH* | 5’-ACATCGCTCAGACACCATG-3’ | 5’-TGTAGTTGAGGTCAATGAAGGG-3’ |

**Supplementary Figure**

**
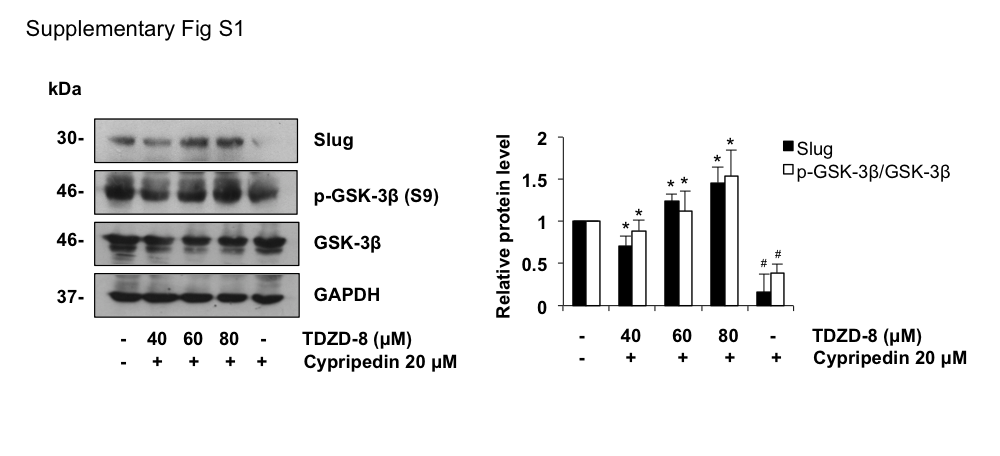
**

**Figure S1** Cypripedin decreased Slug expression via GSK-3β activation. H460 cells were pretreated with a selective GSK-3β inhibitor, TDZD-8 (40-80 µM), prior to incubation with cypripedin (20 µM) or DMSO for 24 h. Cell lysate were collected and analyzed for Slug, GSK-3β and p-GSK-3β expressions by Western blotting. The protein intensity was qualified by densitometry relative to GAPDH. The data are presented as mean ± SEM (n=4). ^#^ *p* < 0.05 compared with non-treated cells. * *p* < 0.05 compared with cypripedin-treated cells.


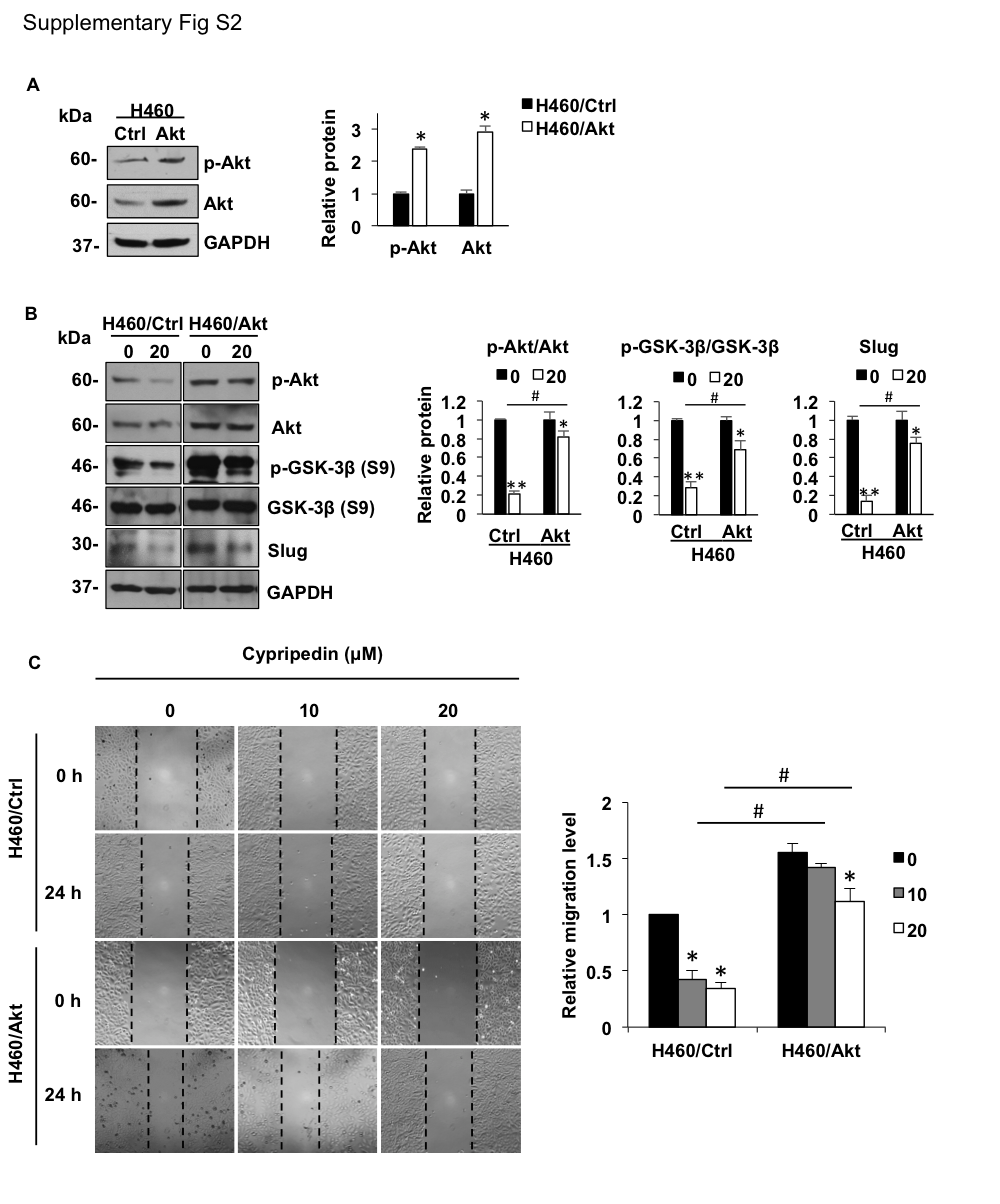


**Figure S2** Akt overexpression confers cypripedin-suppressing EMT. H460 cells were transfected with active Akt plasmid or pcDNA3 as control. (A) Akt and p-Akt expressions in transfected H460 cells. **p* < 0.05 compared with H460/Ctrl cells. (B) Transfectants were treated with or without cypripedin (20 µM) for 72 h, and analyzed for p-Akt, Akt, p-GSK3β, GSK3β and slug. The protein levels were qualified by densitometry relative to GAPDH. The data are presented as mean ± SEM (n=4). **p* < 0.05 and ** *p* < 0.01 compared with non-treated cells. ^#^ *p* < 0.05 compared with cypripedin-treated H460/Ctrl cells. (B) Under similar treatment, cell migration assay was performed. The wound space was captured and measured at 0 and 24 h. The wound area was calculated and presented as a relative value to the area at the initial time point. The data are presented as mean ± SEM (n=4). **p* < 0.05 compared with non-treated cells. ^#^ *p* < 0.05 compared with H460/Ctrl cells.

**
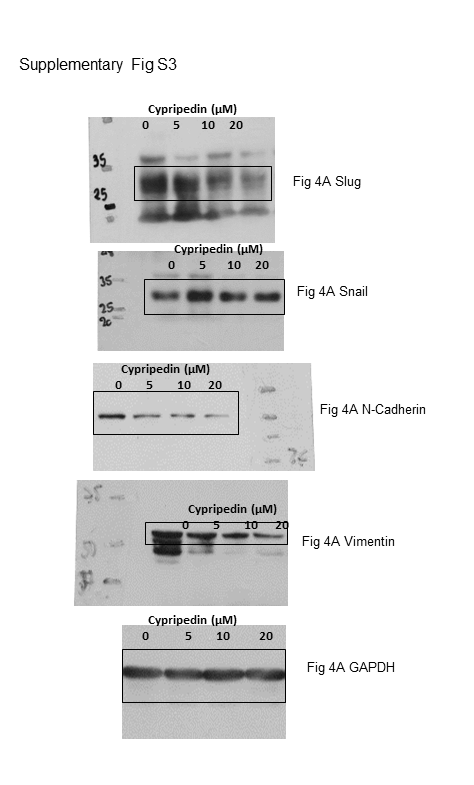
**

**Figure S3** Uncropped western blot used in Fig 4A

**
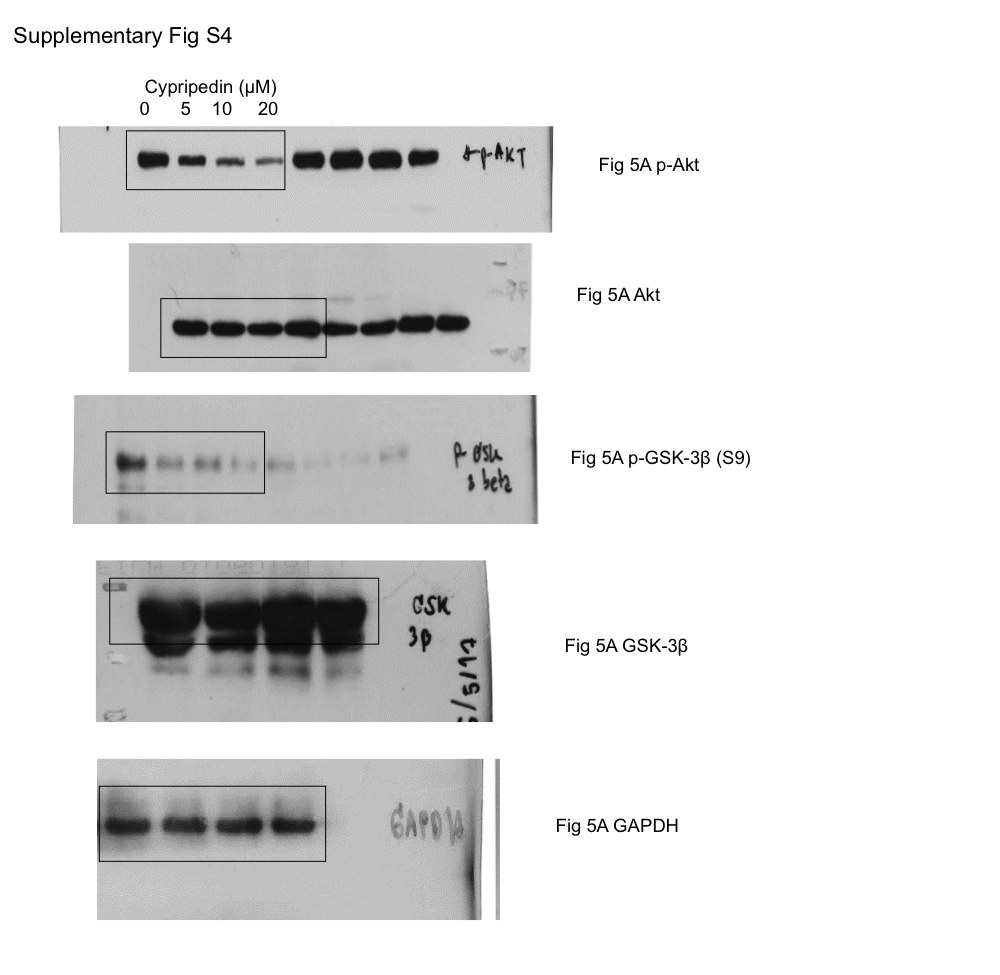
**

**Figure S4** Uncropped western blot used in Fig 5A

**
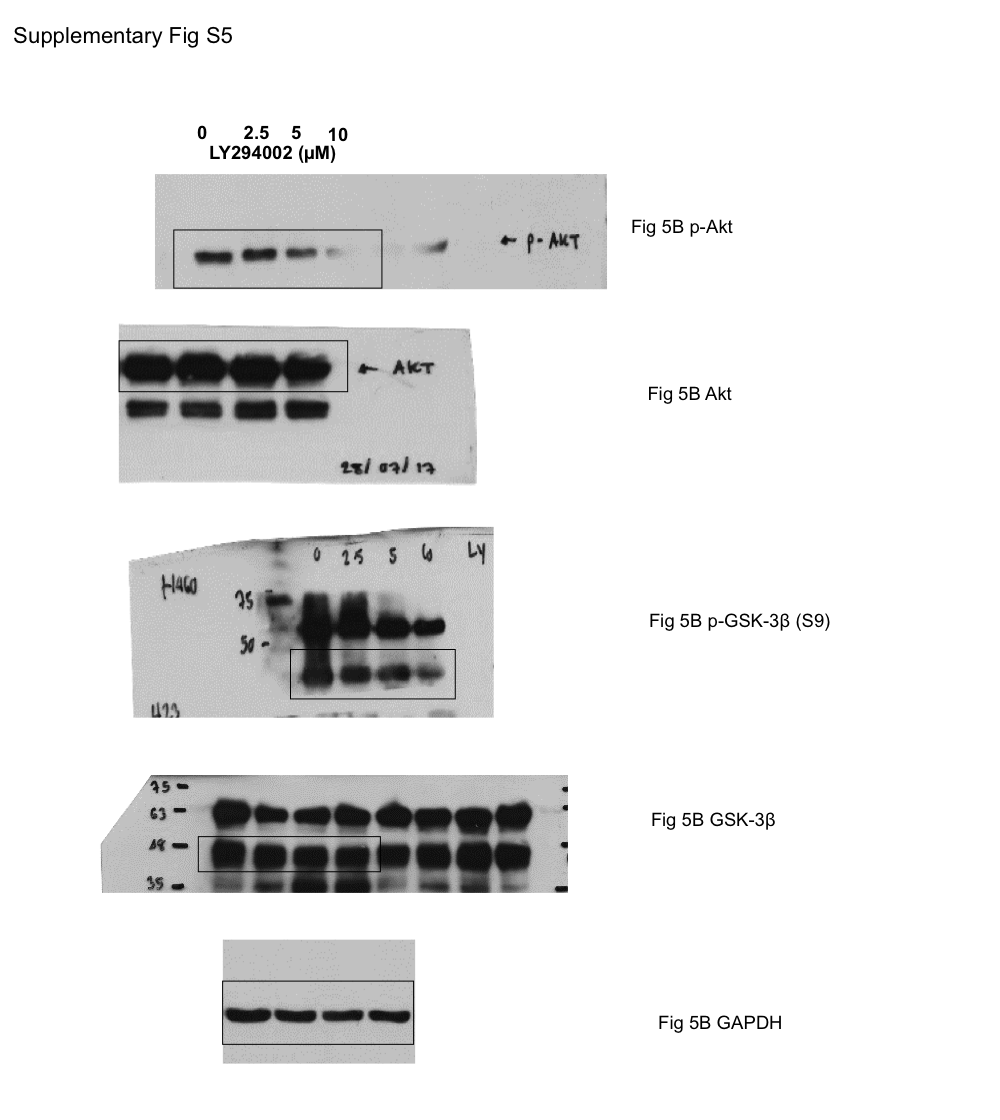
**

**Figure S5** Uncropped western blot used in Fig 5B

**
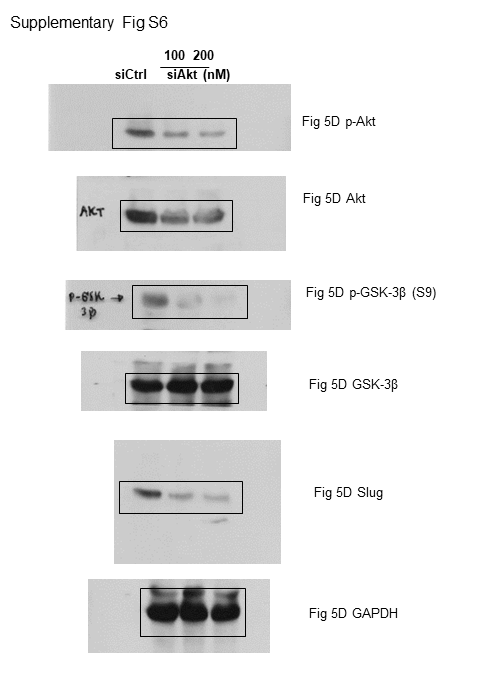
**

**Figure S6** Uncropped western blot used in Fig 5D

**
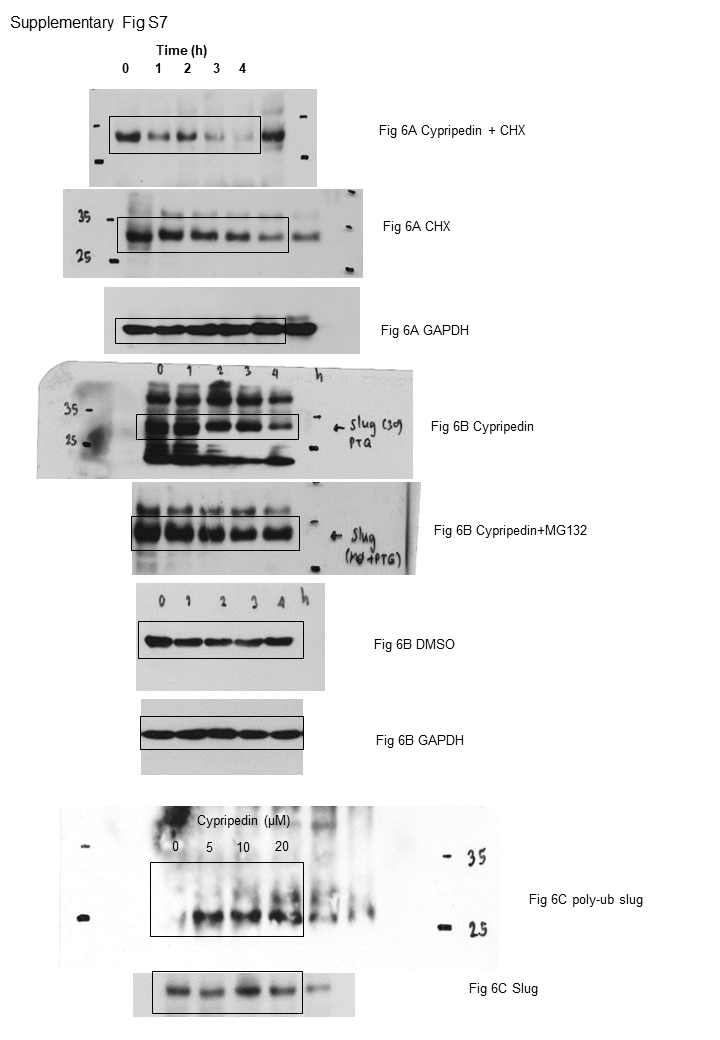
**

**Figure S7** Uncropped western blot used in Fig 6

**
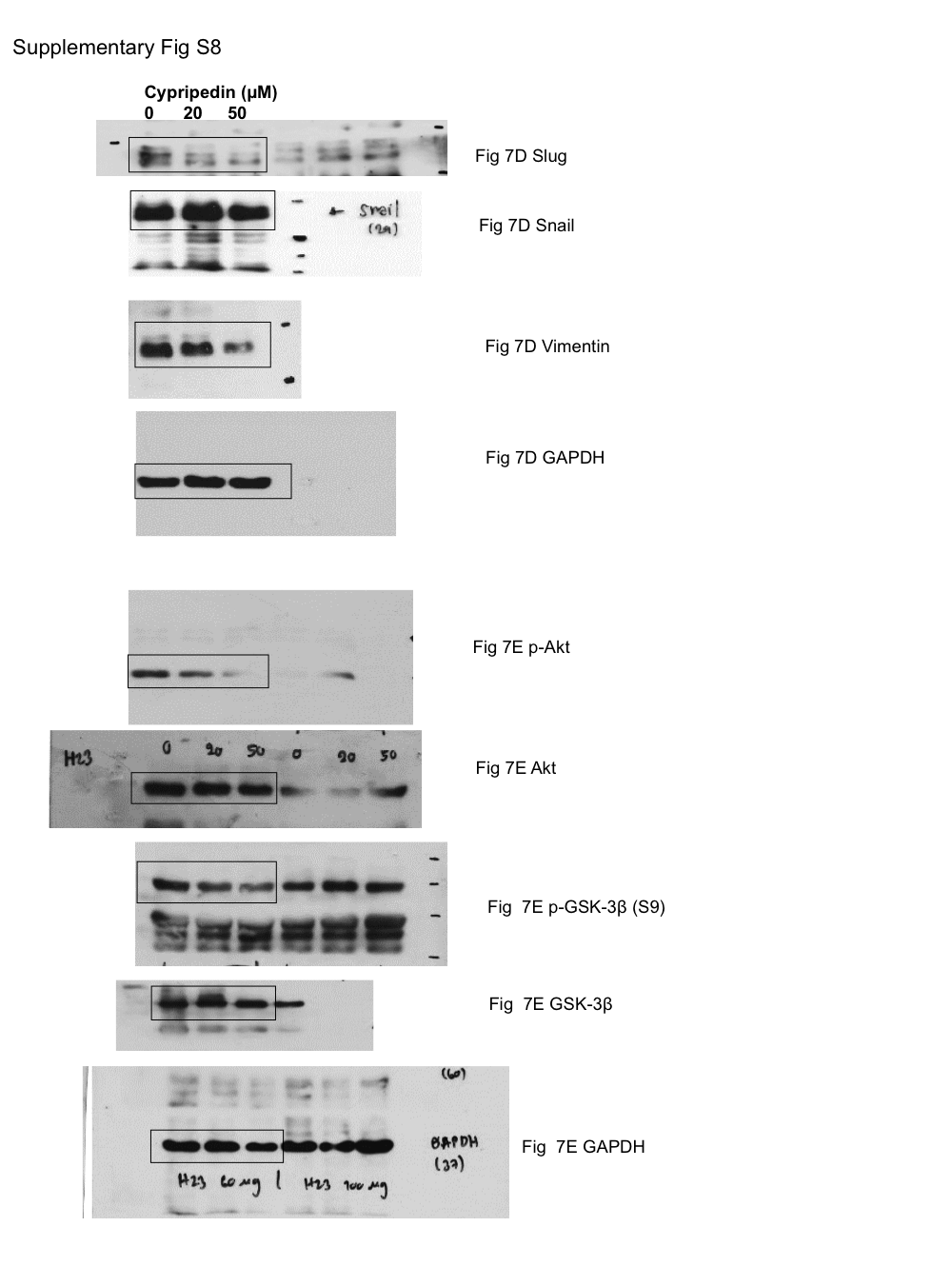
**

**Figure S8** Uncropped western blot used in Fig 7

**
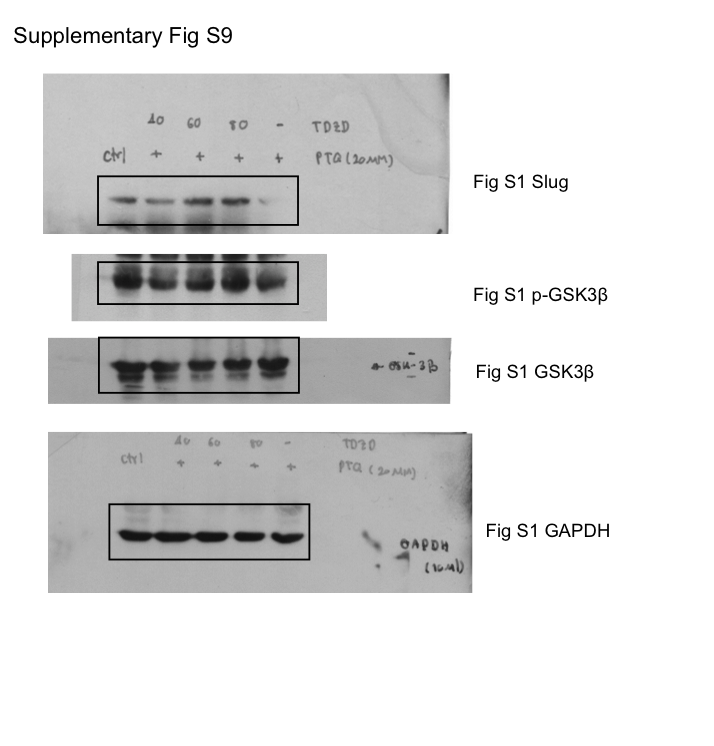
**

**Figure S9** Uncropped western blot used in Fig S1

**
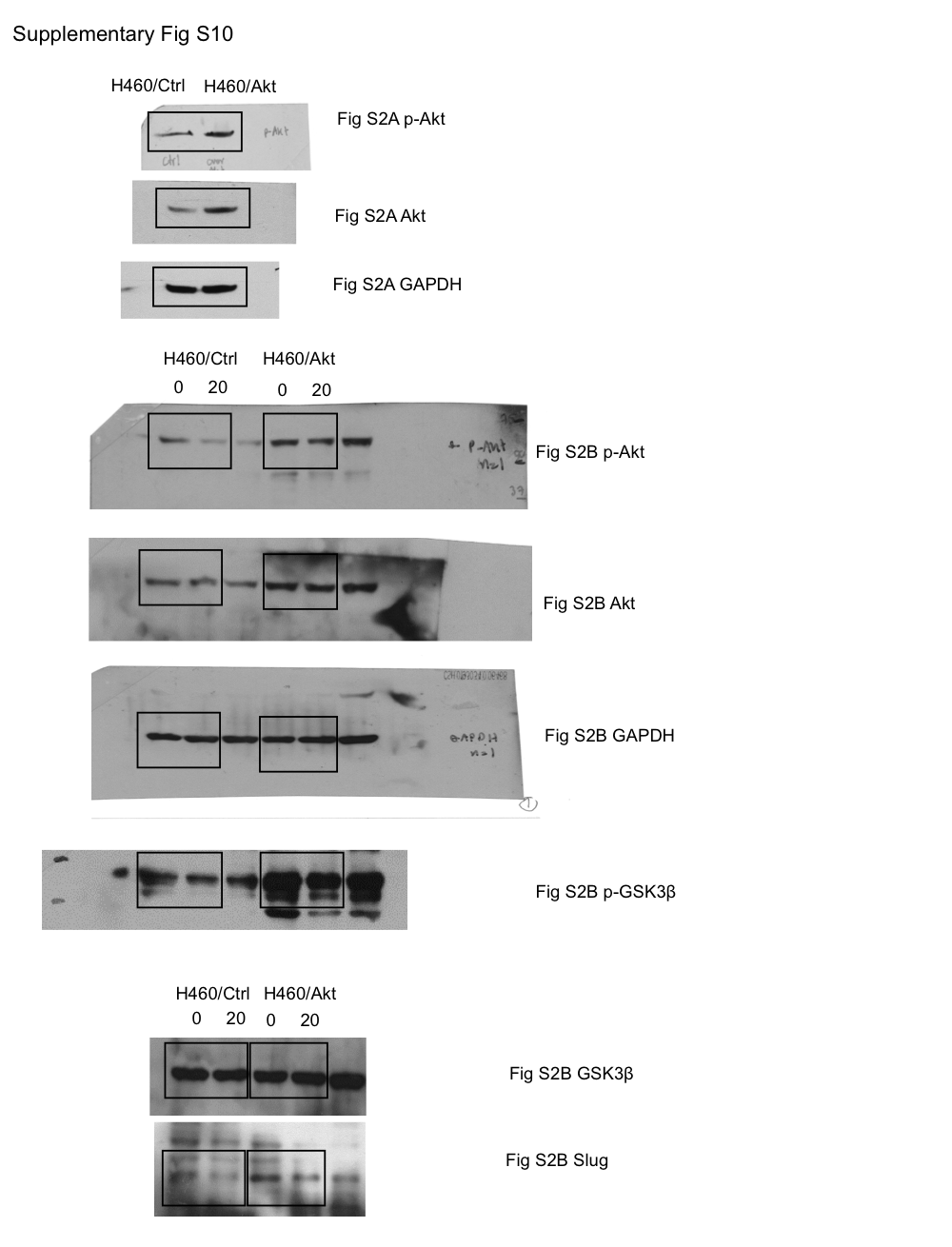
**

**Figure S10** Uncropped western blot used in Fig S2
